# Reduction of 5G cellular network radiation in wireless mobile phone using an asymmetric square shaped passive metamaterial design

**DOI:** 10.1038/s41598-021-82105-7

**Published:** 2021-01-29

**Authors:** Tayaallen Ramachandran, Mohammad Rashed Iqbal Faruque, Air Mohammad Siddiky, Mohammad Tariqul Islam

**Affiliations:** 1 Space Science Center (ANGKASA), Universiti Kebangsaan Malaysia, 43600 Bangi, Selangor Malaysia; 2 Department of Electrical, Electronic and Systems Engineering, Universiti Kebangsaan Malaysia, 43600 Bangi, Selangor Malaysia

**Keywords:** Electrical and electronic engineering, Electronic properties and materials

## Abstract

This study aims to demonstrate the feasibility of metamaterial application in absorption reduction of 5G electromagnetic (EM) energy in the human head tissue. In a general sense, the radio frequency (RF) energy that received by wireless mobile phone from the base station, will emit to surrounding when the devices are in active mode. Since the latest fifth generation technology standard for cellular networks is upon us, the emission of radiation from any wireless devices needs to be taken into consideration. This motivation helps to prepare this paper that focuses on construction of novel and compact square-shaped metamaterial (SM) design to reduce electromagnetic exposure to humans. The commercially available substrate material known as FR-4 with thickness of 1.6 mm was selected to place the metamaterial design on it. The electromagnetic properties and Specific Absorption Rate (SAR) analyses were carried out numerically by utilising high-performance 3D EM analysis, Computer Simulation Technology Studio (CST) software. Meanwhile, for the validation purpose, the metamaterial designs for both unit and array cells were fabricated to measure the electromagnetic properties of the material. From the numerical simulation, the introduced SM design manifested quadruple resonance frequencies in multi bands precisely at 1.246 (at L-band), 3.052, 3.794 (at S-band), and 4.858 (C-band) GHz. However, the comparison of numerically simulated and measured data reveals a slight difference between them where only the second resonance frequency was decreased by 0.009 GHz while other frequencies were increased by 0.002, 0.045, and 0.117 GHz in sequential order. Moreover, the SAR analysis recorded high values at 3.794 GHz with 61.16% and 70.33% for 1 g and 10 g of tissue volumes, respectively. Overall, our results demonstrate strong SAR reduction effects, and the proposed SM design may be considered a promising aspect in the telecommunication field.

## Introduction

The latest 5G telecommunication network is finally upon us. This network is faster and capable of managing more connected devices than the currently available 4G network. Although the 5G network has been highly recommended to comply with current technological advances, it possesses several disadvantages. The radiation exposure from the mobile phone may cause serious health problems such as skin disease, brain tumour, heart disease, irregular blood pressure, sleep disorder, or even cancer^1–4^. Based on the International Commission on Non-Ionizing Radiation Protection (ICNIRP) and the IEEE C95.1-2019 standards, the SAR limit is set to 2 W/kg for the averaged over 10 g of tissue volume^5,6^. Many telecommunication companies are investigating the potential methods to reduce the SAR value that is exposed to the mobile phone users. For this reason, it was of interest to investigate the reduction of the SAR values at the referred frequency range.

Based on these guidelines, the method to reduce the EM radiation at desired lower resonance frequencies, becomes a challenging issue among scientists. In recent years, a metamaterial design structure is becoming a popular topic in the telecommunication field and the material possesses an exotic and outstanding performance. In general, this man-made material is not found in nature but has unique electromagnetic properties that are not possible in conventional materials. Metamaterial widely used in many research fields besides the SAR reduction application, namely optical cloaking, microwave application, satellite application, metasurface, and absorber^7–14^. This paper begins with a short review of the literature regarding the stated application fields which are heterogeneous to one another, but the unique properties of the unconventional material enables the scholars to obtain extraordinary findings. Hence, at this present time, numerous research works have been carried out in the telecommunication field to examine the ideal SAR reduction values.

Prior research studies suggest that precise metamaterial design structure has potential to influence the SAR values that are simulated through any related computer software. In 2009, Manapati et al.^15^ proposed a single negative metamaterial design to reduce the EM interaction between mobile phone and human head. The metamaterial structure was designed from periodic arrangement of open and spiral split-ring resonators (SRR). A multilayer planar inverted-F antenna (PIFA) with electromagnetic band-gap (EBG) was introduced by Kwak et al.^16^ to minimise the EM radiation from the mobile phone. Meanwhile, Hwang et al.^17^ suggested square-shaped metamaterial design to reduce undesired radiation at 0.90 and 1.80 GHz respectively. The authors effectively reduce the SAR values by utilising finite-difference time-domain method during the simulation process. Studies of Faruque et al.^18,19^ are well documented and it is also well acknowledged that both works were focused on SAR reduction application in separate years respectively. One of the studies^18^, focused on selected frequency range from 0 to 2.10 GHz and the authors proposed square-shaped metamaterial to calculate SAR values in muscle cube. Meanwhile in^19^, Faruque developed a compact sized, double-negative triangular metamaterial structure for the specified application. Furthermore, the scattering parameters of the proposed metamaterial design were calculated for frequency range from 0 to 1.20 GHz. In 2011, Ikeuchi et al.^20^ suggested a dipole antenna above the EBG substrate material. The focus of the authors is to cover 4G wireless communication system and successfully manifested resonance frequency at 3.50 GHz. Whereas Vuchkovikj et al.^21^ numerically simulated the SAR values with diverse positions of inhomogeneous and homogeneous human models, which are commonly known as HUGO and Specific Anthropomorphic Mannequin (SAM) models, respectively. This research study exclusively targeted a frequency range from 0 to 2.50 GHz but has just limited two resonance frequencies which were at 0.90 and 1.80 GHz.

Despite the fact that there are various research works that were performed in the SAR reduction field, they remain limited in terms of Global System for Mobile Communications (GSM) resonance frequencies and their performance. For instance, Janapala et al.^22^ in 2019 proposed a polydimethylsiloxane-based flexible antenna metasurface. In this research work, the authors analysed two different antennas with leaf structure design, i.e., with and without metasurface at 2.40 GHz WLAN ISM band. Besides that, a numerical simulation for SAR reduction applications that have just two resonance frequencies at L- and S-bands, respectively were investigated by Ramachandran et al.^23^. A 11 × 11 mm^2^ and 1.60 mm thick dielectric substrate material was selected for this study with employed 0.30–4 GHz frequency range. Meanwhile, in the same year, Chaudhary and Vijay^24^ examined the consequences of antenna position that placed near to the human head on calculated SAR values. The study mainly focused on the construction of dielectric shielding material to increase the SAR reduction percentage value that is absorbed by the human head. This aim was successfully achieved by the analyses of various antenna positions inside the mobile phone and diverse material shields. Furthermore, an inclusive research work can be found in^25^, in which Stephen et al. investigate the effects of EM fields on humans and effectively lowered the exposed SAR values in human tissue cube for biomedical application. In this research work, the authors also included the reviews of diverse EM shielding materials applications such as ferrite materials, conductive materials, dielectric materials, and metamaterials. In 2017, Pandey and Singhal^26^ studied the effects of metamaterial structure design on SAR value with varied locations, sizes and distances at 0.90 GHz resonance frequency. Pandey et al. constructed a circular SRR on the 1.60 mm thick FR-4 substrate material.

In 2016, Rosaline et al.^27^ and Dutta et al.^28^ investigated the single SRR superstrate and diverse shielding material for the SAR reduction application. In^27^, the superstrate manifested dual resonance frequencies with a primary peak point occurred at 0.90 GHz. However, both research works have one familiar correspondence whereby they produced a similar resonance frequency of 1.80 GHz. Furthermore, both studies utilised distinct simulation software (i.e., CST and ANSYS HFSS) and exhibited varying numbers of resonance frequency. Besides that, the SAR values were successfully computed by utilising respective software. Mat et al.^29^ numerically simulated the SAR values that were computed inside the human head with an ear implantation. The ear prosthesis was made up of a metallic bar and placed at the same location as the real human ear. This research work can manifest multiple resonance frequencies, namely 0.90 GHz, 1.80 GHz, and 2.10 GHz. Meanwhile, a SAR investigation for four different age groups of anatomical head models were conducted by Lee et al.^30^. The results then compared with the numerical simulation data obtained through SEMCAD X software by adapting the SAM phantom model and typical bar-type mobile phone. However, the study focused on the two resonance frequencies only, namely 0.835 GHz and 1.850 GHz.

Despite the fact that, earlier studies stated in the literature review have significant contribution in reducing the SAR value that emitted from the wireless devices by utilising metamaterial structure. Consequently, previous research works suffer from certain weaknesses although successfully manage to minimise SAR reduction value at distinct resonance frequencies. For instance, necessary improvements are required to protect humans from the radiation exposure of 5th generation wireless communication technology. The standard guidelines of ICNIRP and IEEE will ensure the mobile phone companies and network service providers do not exceed the maximum permissible exposure level although operating in the highest possible condition. Additional studies to understand more completely the key tenets of the 5G network effects and how to prevent the consequences, are required. Emerging latest 5G technology demands an advanced method or design to further reduce the EM radiation effect. Hence, the aim of this research is to maintain the SAR limits by designing a metamaterial shield that can be attached in any mobile phones. Mobile phones with metamaterial design will be tested in the highest power level at all resonance frequencies and in diverse phone positions from the human head. The initial focus of this paper is to demonstrate a compact sized metamaterial design structure that possesses multi-band resonance frequencies and left-handed characteristics.

## Materials and methods

### Unit cell metamaterial construction

**Figure 1:**
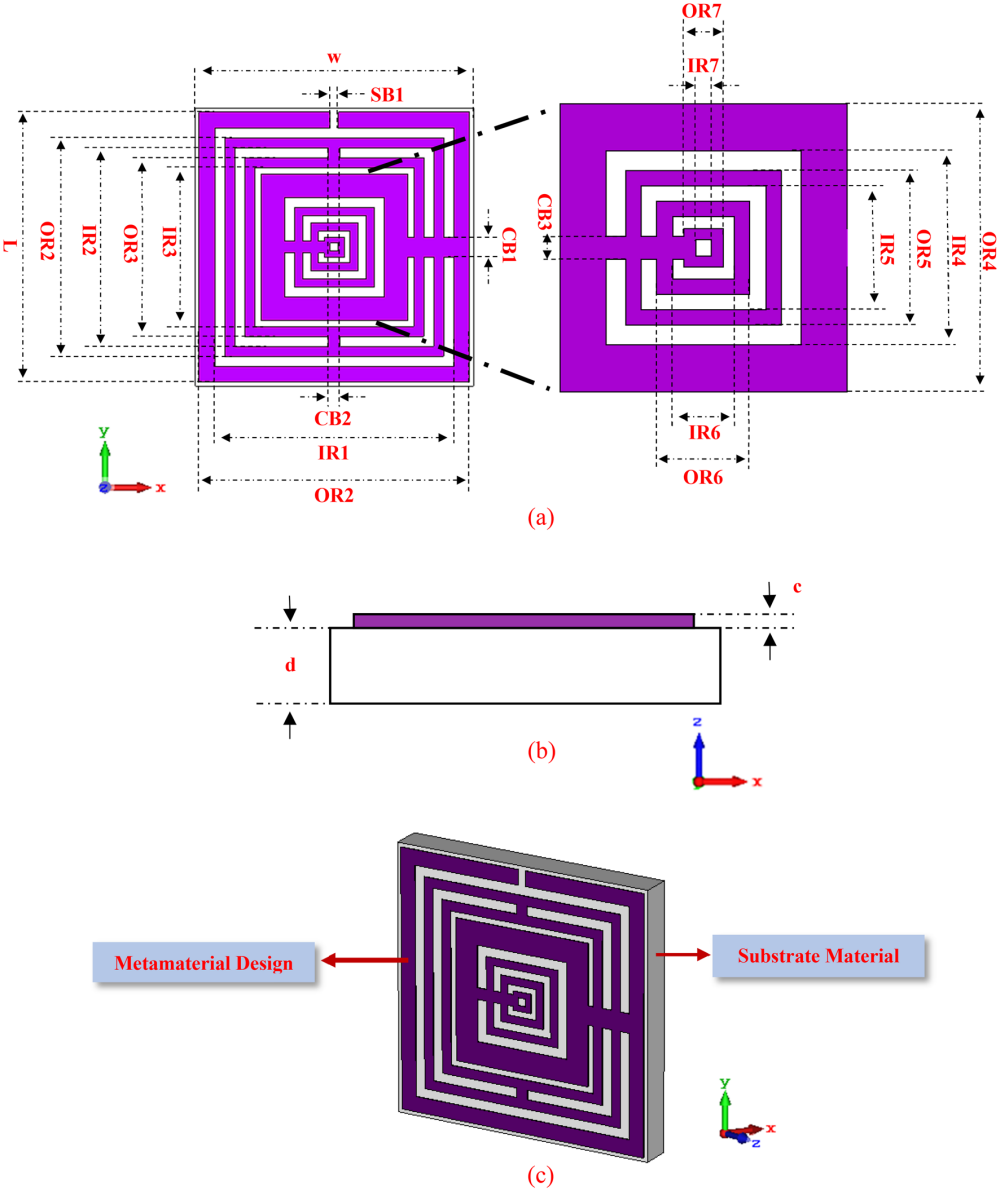
SM design exported from CST software: (**a**) orthographic view from top, (**b**) orthographic view from front, (**c**) isometric projection of overall design.

The initial step for the construction of a suitable design structure was started with the unit cell. The optimised metamaterial design structure will greatly influence the electromagnetic properties and SAR reduction values. Therefore, in the preliminary state, the construction of metamaterial unit cell plays an important role in obtaining the desired resonance frequencies. The metamaterial structure is composed of FR-4 substrate material with thickness (d) of 1.6 mm and has a length (a) and width (b) of 14 mm, respectively. This specific substrate material has dielectric constant (ε) and tangent loss (δ) of 4.3 and 0.025. Overall, seven square rings with designated gaps and splits were constructed on the dielectric substrate material. Meanwhile, the metamaterial structure was designed using annealed copper material which has a conductivity value of 5.80 × 107 S/m. The thickness of 0.035 mm was selected to design the copper material on the surface of FR-4 substrate. The Fig. 1a,b illustrate the exported CST top and front views of the proposed metamaterial design. The square rings were arranged with uneven thickness. The first ring near to the x-axis, was designed with thickness of 1.6 mm and left a gap of 0.5 mm until the second ring. A rectangular bar with width and length (WL) of 0.4 mm and 1.6 mm, was subtracted from the top of the first square ring design. Meanwhile, the next two rings are designed with similar thickness of 1.0 mm and have 0.5 mm gap between them. Rectangular bars with W of 1.0 mm were utilised to connect these both rings at the top and bottom design structure. The 4th ring with a thickness of 2.4 mm was constructed with 0.3 mm gap from the previous ring. Rectangular bar with W of 1.0 mm was placed on the left side of the design structure to connect all the first four rings. Furthermore, the 5th and 6th rings with the same thickness of 0.8 mm were added after a 0.5 mm gap and have 0.4 mm intervals between them. Finally, the last ring was constructed with a thickness of 0.6 mm after an interval of 0.3 mm. A rectangular bar with W of 0.6 mm was added on the left side of the design structure to connect the 5th to 7th rings. The isometric projection view of the complete structure is illustrated in Fig. 1c. The entire dimension details of the proposed unit cell SM design are described in Table 1.

**Table 1:** Dimension details of the proposed SM design.

Descriptions	Dimension (mm)
Outer ring, OR1	13.60
Inner ring, IR1	12.00
Outer ring, OR2	11.00
Inner ring, IR2	10.00
Outer ring, OR3	9.00
Inner ring, IR3	8.00
Outer ring, OR4	7.40
Inner ring, IR4	5.00
Outer ring, OR5	4.00
Inner ring, IR5	3.20
Outer ring, OR6	2.40
Inner ring, IR6	1.60
Outer ring, OR7	1.00
Inner ring, IR7	0.40
Substrate bar, SB1	0.40
Connecting bar, CB1	1.00
Connecting bar, CB2	0.60
Connecting bar, CB3	0.60
Substrate length, a	14.00
Substrate width, b	14.00
Copper thickness, c	0.035
Substrate thickness, d	1.60

### Numerical methods for retrieval of electromagnetic properties and SAR simulations

**Figure 2:**
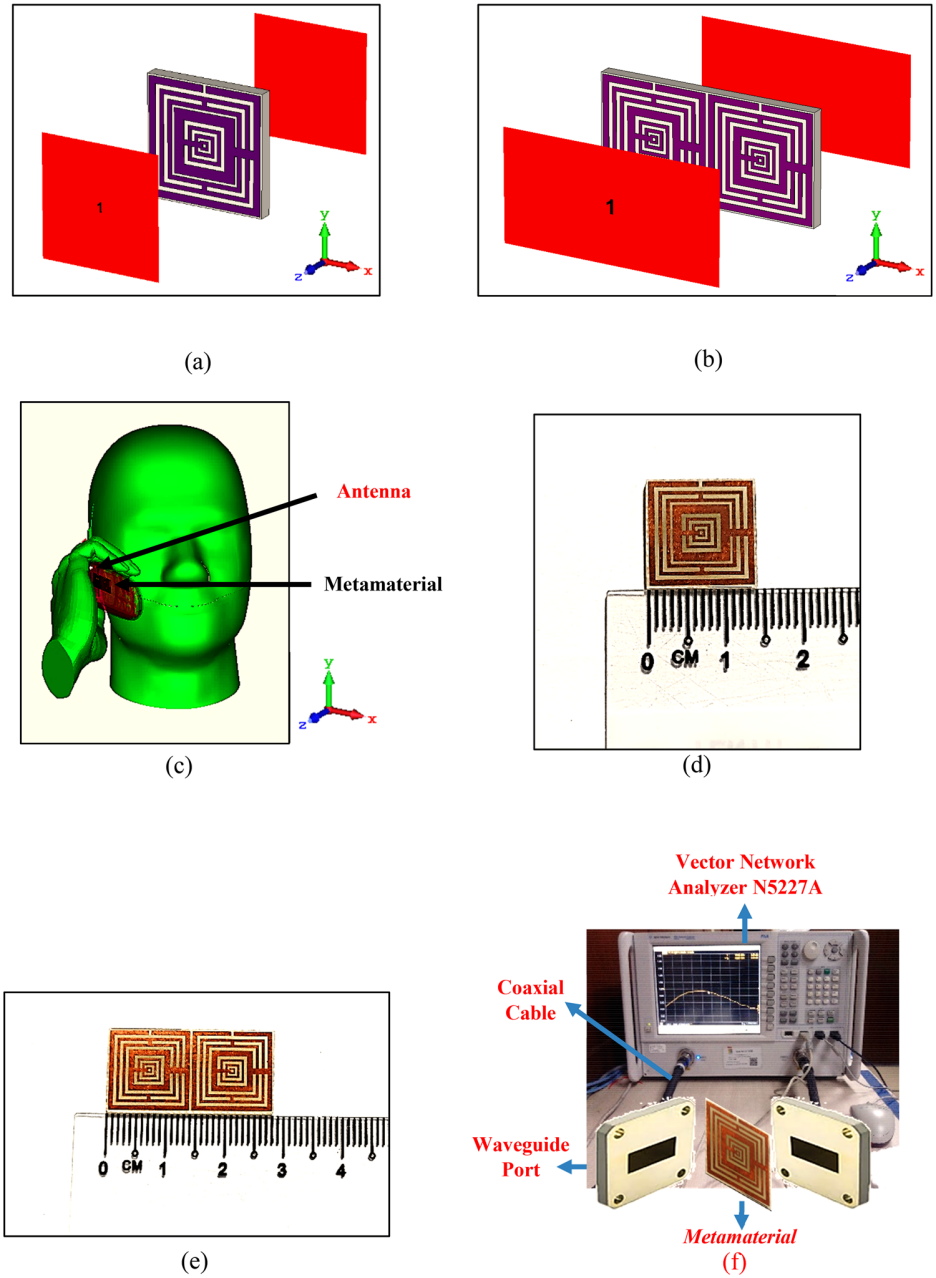
Numerical simulation, fabrication, and measurement: (**a**) unit cell SM metamaterial, (**b**) 1 × 2 array SM metamaterial, (**c**) fabricated unit cell SM metamaterial, (**d**) fabricated 1 × 2 array SM metamaterial, (**e**) voxel human model, (**f**) metamaterial measurement setup of Vector Network Analyzer N5227A.

The numerical simulations in this study were composed of three different stages. All the simulations were performed by utilising well known Computer Simulation Technology (CST) Microwave Studio software. CST software has quick and precise responses for electromagnetic problems solving. The first two simulation stages were focused on the unit and array cells construction and obtained electromagnetic properties of the proposed metamaterial design. Once a unit cell metamaterial design was constructed, then the design was numerically simulated using frequency-domain solver and terahertz mesh in CST. Two waveguide ports were placed in front and back of the metamaterial design structure as demonstrated in the Fig. 2a,b. The ports were set at positive and negative z-axis and utilised Transverse Electromagnetic wave (TEM) mode. Meanwhile, for the boundary condition, the x-axis was defined as Perfect Electric Conductor (PEC), while y-axis was defined as Perfect Magnetic Conductor (PMC). 5G telecommunication network categorised into two frequency ranges, namely Frequency Range 1 (FR1) and Frequency Range 2 (FR2). The FR1 includes sub-6 GHz frequency bands, while FR2 is for communication at the millimeter (mm) wave frequencies above 24 GHz, respectively. In this paper, the FR1 category is examined for the SAR reduction application. Therefore, the frequency range from 0 to 5 GHz was selected to satisfy the objective of this research study. The scattering parameters obtained through the initial simulation were used to calculate the effective medium parameters of the metamaterial design. These parameters were obtained by using MATLAB^31–33^ software and applied the familiar Robust method. The retrieval equations of impedance (z), refractive index (n), permittivity (ε), and permeability (μ) using the Robust method are defined in Eqs. (1)–(4). Firstly, the MATLAB coding calculates the impedance and refractive index values of the proposed metamaterial design by utilising the Eqs. (1) and (2), where the signs ′ and ″ described real and imaginary, respectively. Meanwhile, the permittivity and permeability values were directly calculated from Eqs. (3) and (4).

1 $$z=\pm \surd \frac{{(1+{S}_{11})}^{2}-{S}_{21}^{2}}{{(1-{S}_{11})}^{2}-{S}_{21}^{2}}$$

2 $$ n = \frac{1}{{k_{0} d}}\left\{ {\left[ {In\left( {e^{{ink_{0} d}} } \right)} \right]^{\prime \prime } + 2m\pi - i\left[ {In\left( {e^{{ink_{0} d}} } \right)} \right]^{\prime } } \right\},\quad e^{{ink_{0} d}} = \frac{{S_{21} }}{{1 - S_{11} \frac{z - 1}{{z + 1}}}} $$

3 $$\varepsilon =\frac{n}{z}$$

4 $$\mu =nz$$

The final stage in the simulation process was the SAR reduction calculation to determine the impact of precise metamaterial design structure inside the mobile phone. CST Studio Suite can directly calculate all types of specific absorption rate (SAR), including point SAR, 1 g and 10 g-average SAR and whole-body SAR, and contains a Multiphysics module with bio-heat solvers that can calculate temperature distributions including effects of living tissues such as metabolic heating, blood diffusion and human thermoregulation. For this purpose, the Specific Anthropomorphic Mannequin (SAM) Phantom was adapted from the CST component library. Typically, this simplified phantom model is homogenous to the real human head which follows IEEE standards. This phantom model is made up of two parts, for instance an outer shell and it is filled with tissue simulating liquid. Generally, this model was constructed based on 90% of the male population’s head size to standardise the certification. A simple flip mobile phone model was used for this analysis. The mobile phone placed on the right-hand side of the human head with the hand model holds it in talk mode. The satisfied metamaterial design then placed inside the mobile phone to calculate the SAR reduction values. The full setup of SAR reduction simulation is illustrated in Fig. 2c. Meanwhile, the phone model was made up of LCD components, keyboard, circuit, antenna, and housing. Table 2 presents the dielectric properties of the human head model, and phone model. The dielectric properties of the SAM head model gained through the properties of tissues inside the anatomical head. Therefore, the outer shell possesses frequency independent permittivity value of 3.7 and an electrical conductivity of 0.0016 S/m. On the other hand, the tissue simulating liquid has frequency dependent dielectric properties. Their values of four resonance frequencies are as well illustrated in the Table 2.

**Table 2:** Dielectric properties of the phone material and SAM phantom.

	ε_r_	σ (S/m)
**Phone parts and materials**		
LCD glass	4.78	–
LCD cover pad	3.50	0.005
Circuit PCB	4.90	–
Housing	2.50	0.005
Keyboard	3.50	0.005
**SAM phantom**		
Head shell	3.70	0.0016
Liquid @ 1.2 GHz	41.47	0.98
Liquid @ 3.0 GHz	38.72	2.43
Liquid @ 3.7 GHz	38.72	2.43
Liquid @ 5.2 GHz	36.00	4.70

The proposed metamaterial was placed inside this mobile phone model without distressing any existing components. The manifested resonance frequencies of the array cell SM design were fixed in the simulation setting. Then, the SAR numerical simulation was successfully performed by utilising a time-domain solver. The right cheek of the human head and antenna structure were aligned together at the centre point. The surface of the head model is not necessarily flat when a user holds the phone in talk mode. Hence, approximately 20 mm distance was left between the antenna and human head. The excitation of the discrete face port is defined as 50 Ω; and the frequency range is between 0 and 5 GHz. Moreover, in this simulation analysis, a default peak reference power of 0.50 W was applied, allowing the power to flow directly into the metamaterial structure. As the reflection is lost at the feeding spot, the SAR calculation is not affected by the default power. Basically, the radiated power of an antenna acts like a reference power in the absence of the ohmic loss condition. Thus, for the SAR calculation averaged over 1 g and 10 g of tissue volumes, power-loss density was adapted in this simulation.

### Measurement

The measurement process was only carried to validate the electromagnetic properties of the proposed metamaterial design in this research study. The Fig. 2d,e show the fabricated SM design structure of the unit and array cells, respectively. The printed circuit board (PCB) was utilised for the fabrication process. The manufactured FR-4 PCB board typically comes together with a copper layer printed on both sides. Based on the proposed metamaterial, the rest of the copper was removed from the surface of FR-4 material. The well-known vector network analyser (VNA) model number of Agilent N5227A was used to measure this fabricated metamaterial design. The illustration of the measurement setup is demonstrated in Fig. 2f. Both unit and array cells were placed between two waveguide port cables from VNA, discretely to measure the scattering parameters. Three types of adapters were adapted in this study, namely A-INFOMW WG to adapter P/N: 510WCAS for L-band, A-INFOMW WG to Coaxial adapter P/N: 187WCAS for S-band and A-INFOMW WG to adapter P/N: 137WCAS for C-band. Due to the specific conditions, the hardware can cause an error during the measurement. Hence, to obtain the accurate readings, an Agilent N4694-60001 device was utilised to calibrate VNA prior to the measurement process began.

## Discussion

### Metamaterial electromagnetic properties

**Figure 3:**
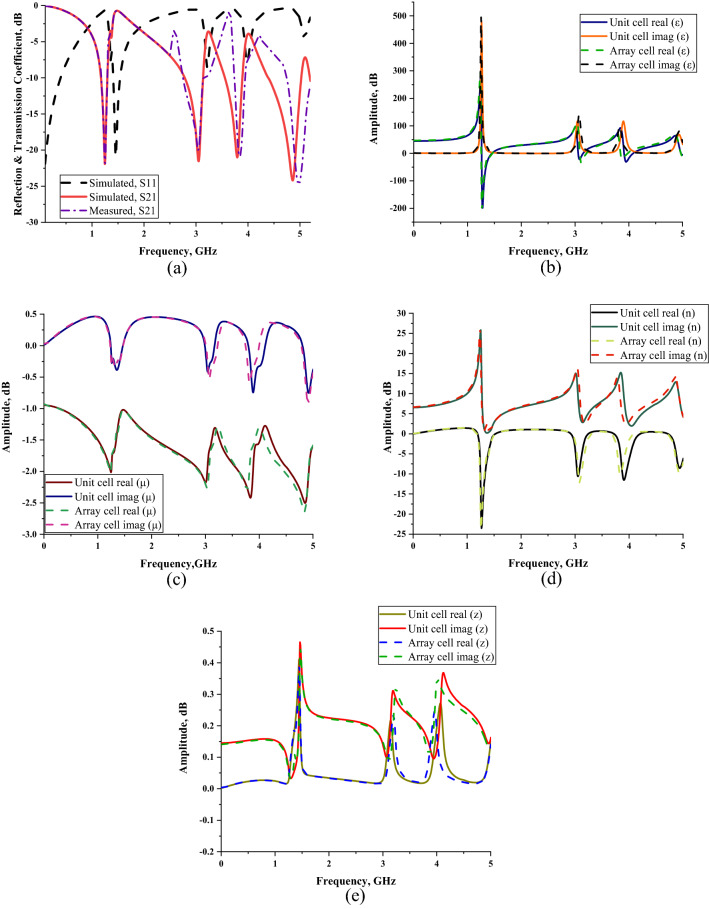
Scattering parameter of the proposed SS metamaterial for array cell: (**a**) numerically simulated (S11 and S21) and measured (S21); effective medium parameters for both unit and array cells (**b**) permittivity, (**c**) permeability, (**d**) refractive index, (**e**) impedance values.

Figure 3a–e demonstrate the results of the scattering and effective medium parameters that obtained through the numerical simulation. From the short review above, key findings emerge that, proposed SM design manifest multi-band resonance frequencies at L-, S-, and C-band, for instance 1.246, 3.052, 3.797 and 4.858 GHz with suitable magnitude values of − 21.933, − 21.556, − 21.019, and − 24.212 dB, respectively. The Fig. 3a exemplifies the reflection coefficient (S11) of the numerical simulation and transmission coefficient (S21) of both numerical and measurement methods. Another finding indicates that, there was a slight difference occurred among the resonance frequencies between numerical and measurement results. The measurement data manifests peak resonance at 1.248, 3.043, 3.842 and 4.975 GHz. Meanwhile, this method as well exhibits tolerable magnitude values of − 22.303, − 20.005, − 20.899, and − 24.943 dB, respectively. It is worth discussing these interesting facts revealed by the results of both methods, these slight discrepancies probably provoked by several elements. We speculate that this might be due to the calibration error that has great influence on the data produced. The measurement device underwent a calibration process by utilising Agilent N4694-60001 Ecal kit before the experiment. This is because the ambient temperature effects can change the measured results. Besides that, the inconsistent results in both methods are also likely caused by the minor errors during fabrication procedure. Since, the SM design is compact in size, intermittently flaws are possible when removing excess copper from the substrate material. The mutual effects that present between the two waveguide ports may cause the subtle variation in results. On that note, approximately 1.012% of mean difference occurred between the two methods for array SM metamaterial design. However, the initial resonance frequency for both simulation and measurement methods indicate similar outcomes. This is because the waveguide port which has frequency range that start from 1.45 GHz was utilised for the measurement of first resonance frequency. On the other hand, the S11 and S21 of the numerical simulation of the array SM design as shown in Fig. 3a, exhibit distinct outcomes. It has something to do with the fact, the entire power is not reflected or transmitted during the simulation.

The Fig. 3b–e illustrate the effective medium parameters of the SM design. This platform helps to distinguish the effect of the metamaterial structure when exposed to external time variant electromagnetic fields. Typically, the relationship of electric and magnetic fields can relate to the physical characteristics of electromagnetic fields. Meanwhile, the material types that are utilised for the metamaterial structure, as well influence the results. Generally, most of the material types fall into two major categories, for instance dispersive and lossy material. These material types help to produce desired resonance frequencies and extraordinary permeability and permittivity values. The permittivity values of the array cell SM design were demonstrated in the Fig. 3b. The negative behaviour occurred at all three resonance bands. Initially, the frequency range from 1.260 to 1.456 GHz (at L-band) has negative values with tolerable below-zero amplitude values of − 154.004 and − 0.339 dB, respectively. Meanwhile, at S-band, two negative behaviours happened from 3.094 to 3.206 GHz and 3.850–3.969 GHz with amplitude values of − 14.502 to − 0.249 dB and − 6.985 to − 0.168 dB, respectively. Finally, the negative behaviour occurred at C-band that has frequency range from 4.970 to 4.998 GHz. At this band, the amplitude values recorded the lowest when compared to other three frequency ranges. However, the amplitude values maintain below zero with range from − 3.083 to − 9.996 dB. The analysis above highlighted that the maximum permittivity value occurred at the frequency of 1.267 GHz with amplitude value of − 205.588 dB. Furthermore, a key finding emerges that the permittivity values of both FR-4 and metamaterial structure have major contributions to obtain desired outcomes. Every resonance frequency that gained from the simulation was dominated by these permittivity values. The resonance frequencies were substantially shifted to lower bands when permittivity values were higher. The capacitance value at interval of radiating element and ground were influenced by the effect of the dielectric constant escalation.

The permeability values for the proposed metamaterial design have negative values from 0 to 5 GHz (as shown in Fig. 3c). The overall permeability values were maintained near zero and had peak value at 4.844 GHz with amplitude value of − 2.636 dB. Furthermore, the analysis of metamaterial parameters reveals another promising highlight where the SM design exhibits left-handed characteristic for all resonance bands. The theory of left-handed characteristic has behaviour that not only the refractive index having negative values, but the permeability and permittivity values need to be below zero as well. The left-handed metamaterial can be practicable for many application fields, namely satellite application, radome, terahertz frequency, antenna, EM absorption reduction, etc. This unique property is commonly used to optimise the outcome by possessing diverse behaviours such as negative refractive index, propagation in backward wave and reversed Doppler shifts. There were four left-handed behaviours recorded in this analysis that manifest one frequency range at least for each band. The similar set of frequency ranges with negative permittivity behaviour were obtained for these left-handed characteristics. Firstly, the characteristic has *µ* and *n* (as illustrated in Fig. 3d) values that manifested from − 1.736 to − 1.035 dB and − 23.695 to − 1.510 dB, respectively. Meanwhile, the next two frequency ranges occurred at S-band that have *µ* and *n* values from − 1.683 to − 1.319 dB, − 11.956 to − 2.889 dB, − 1.662 to − 1.344 dB and − 9.941 to − 2.799 dB, in each instance. Finally, at C-band the SM design exhibits left-handed characteristic with *µ* and *n* values from − 1.612 to − 1.586 dB and − 8.761 to − 7.399 dB, respectively.

Figure 3e illustrates the calculated impedance values of the SM design through MATLAB software. Overall, three peak impedance values were documented in this study at first two resonance bands. The proposed metamaterial design attained a maximum point of 0.391 Ω at 1.449 GHz, while the highest point reached 0.237 and 0.246 Ω at 3.199 and 3.962 GHz, respectively. Extensive data analysis shows that the unit and array cells only have slight differences in effective medium results which most probably can be neglected. Due to the real impedance and imaginary refractive index values demonstrated above-zero points, the proposed SM design can be declared as a passive medium. Overall, our SM design was the one that obtained the most robust results. Tables 3 and 4 demonstrate the electric and magnetic field distributions on SM design of front and back view at all resonance frequencies, respectively. Meanwhile the surface current distribution of the proposed metamaterial was illustrated in the Table 3. The preliminary analysis indicates that a strong electric field distribution (ECF) happened in the entire metamaterial design at all four resonance frequencies. The least ECF concentrated at 4.872 GHz when compared with other three resonance frequencies. Furthermore, on the back side of metamaterial structure, the ECF has strong effects on the first three resonance frequencies. At 4.872 GHz, the ECF has less concentration on the centre position of metamaterial structure. The maximum recorded ECF value was 700 V/m (with high intensity from red decreased to blue colour). Meanwhile, the magnetic field distributions (MCF) have the least effect on the proposed metamaterial. At 1.260 and 4.872 GHz, the SM design exhibits highest MCF distributions compared to other two frequencies. However, the metamaterial structure has least MCF effects on the back side for almost all resonance frequencies. MCF has the highest value of 25 A/m (with high intensity from red decreased to blue colour) in this case study. Nevertheless, we found a common question connected with the exposure of ECF and MCF on the surface of substrate material. Metal-composed materials usually have a free electron within their physical structure. Although the free electron is absent in any dielectric substrate, the ECF and MCF still take place on its surface. We speculate that this might be due to the delocalisation of the electron oscillation present in metal and substrate materials. This enables both phenomena to occur on the FR-4 substrate material. Besides that, the surface current distribution (SCD) at 3.024 GHz recorded the least performance than the other resonance frequencies. At the 1.260 GHz resonance frequency, the proposed metamaterial has SCD performance mostly on the first square ring. In general, the SCD intensity has less effects on SM design at all resonance frequencies. A limit of 513 A/m (with high intensity from red decreased to blue colour) was set for the SCD plots. Overall, our SM design was the one that obtained the most robust results.

**Table 3:**
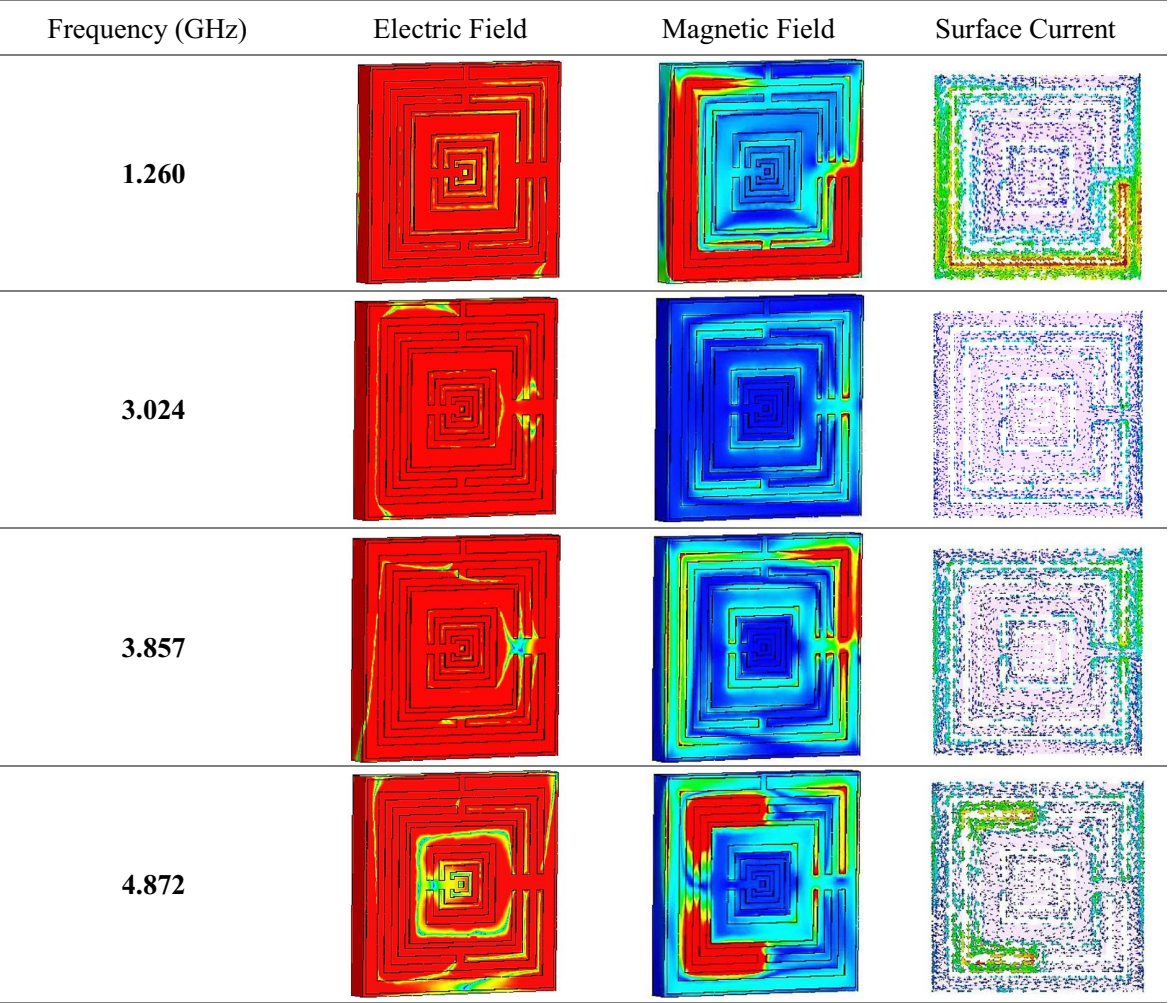
Electric, magnetic field, and surface current distributions of the unit cell SM design for front view.

**Table 4:**
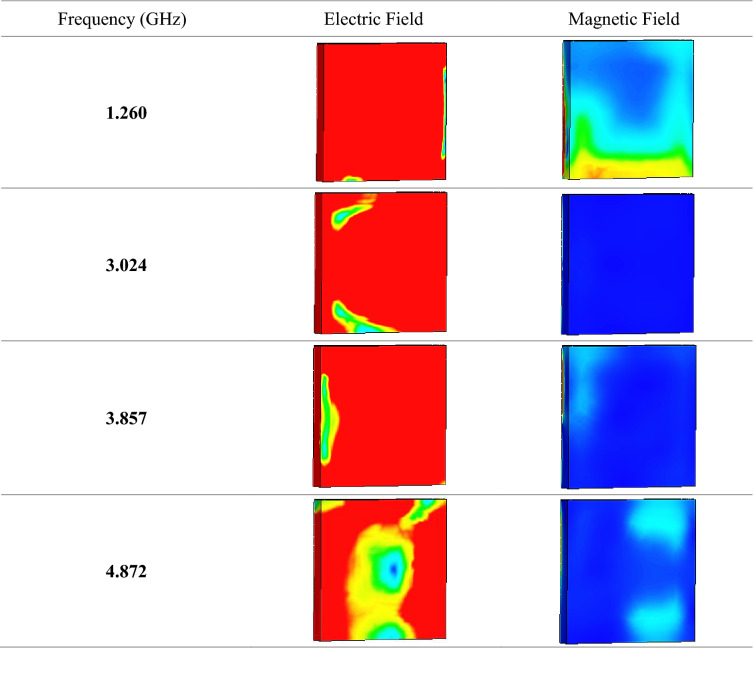
Electric and magnetic field distributions of the unit cell SM design for back view.

**Figure 4:**
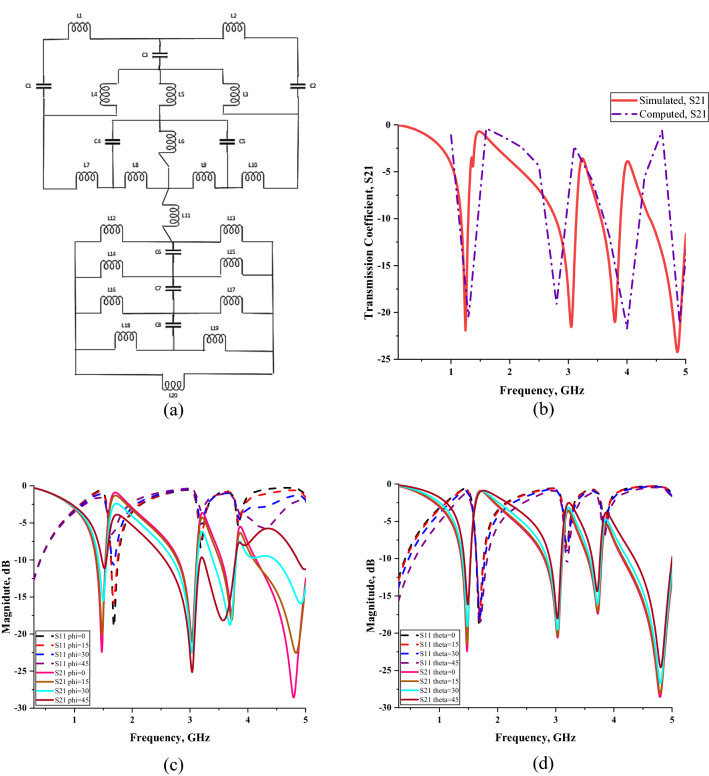
SM design: (**a**) equivalent circuit model, (**b**) comparison of simulated and computed S21 results, (**c**) polarization, (**d**) incident angle.

A circuit model and the computed transmission coefficient result of the SM design are presented in Fig. 4a,b, respectively. The construction of the circuit diagram was successfully plotted by utilising Advanced Design System (ADS) software. The split gaps in this design are adopted as a capacitive effect that is described as C1–C5. Meanwhile the inductive effect is assigned as L1–L20 which clearly indicates that the effect strongly depends on the strip line in the design structure. The transmission coefficient result was computed by using a circuit diagram of the metamaterial structure in ADS and the results were compared with array simulated data. The analysis of transmission coefficient results at second resonance frequency reveal that, the peak value slightly shifted to lower frequency with 8.257% difference. Furthermore, the remaining resonance frequencies were shifted to higher peak values with 0.054, 0.203 and 0.042 GHz difference. Since the comparison of S21 results for both methods have less than 9% discrepancies, hence it can be neglected. Figure 4c,d illustrate that the reflection and transmission coefficients change with the frequency of electromagnetic waves for the polarization, Φ and incident angle, θ from 0 to 45. The simulation was successfully simulated in CST by utilising Phase Reflection Diagram workflow. Both plots clearly indicate that, when the phi or theta increased, then the metamaterial manifests inconsistent magnitude values for all resonance frequency. However, both characteristics product a similar number of resonance frequency with array cell metamaterial. The compactness and effectiveness of the SM design can be calculated using effective medium ratio (EMR) as described in Eq. (5). The EMR formulae defined as wavelength per dimension of the metamaterial design. The results should be more than 4 to satisfy an ideal value. If a metamaterial has ideal EMR value, then the results obtained for permittivity and/or permeability will be less than zero. The proposed SM design has a highest EMR value of 17.20 at the first resonance frequency, which was subsequently reduced for the following resonance frequencies. Overall, slightly superior SAR reduction results were achieved with our compact SM design and it is suitable for EM absorption and reduction application.

5 $$ {\text{EMR}} = {{\left( {{\text{Wavelength }}\left( \lambda \right)} \right)} \mathord{\left/ {\vphantom {{\left( {{\text{Wavelength }}\left( \lambda \right)} \right)} {\left( {{\text{Unit cell length }}\left( {\text{L}} \right)} \right)}}} \right. \kern-\nulldelimiterspace} {\left( {{\text{Unit cell length }}\left( {\text{L}} \right)} \right)}} $$

### SAR reduction

SAR is defined as the rate of radiofrequency energy absorbed by the human body that is exposed from any wireless device. The amount of radiation energy can be calculated by utilising SAR formulas that express in power absorbed per unit mass. The unit of the SAR value is expressed as Watt per Kilogram (W/Kg) and the formula stated in the Eq. (6). The symbols σ is refer to conductivity of material, E to electric field and ${m}_{p}$ to mass density. The SAR values averaged over 1 g and 10 g of tissue volumes of mobile phone without and with metamaterial were numerically simulated by utilising the CST software for all the manifested resonance frequencies of array cell SM design. The Tables 5 and 6 illustrate the human head models that have the maximum SAR values of each frequency and variable using yellow colour contour. SAR analysis clearly indicates that the effect of electromagnetic radiation occurred on the right side of the human head where the mobile phone was placed. Moreover, we discovered that the SAR values average over 1 g of tissue volume have slightly higher reduction percentage values than 10 g, with a reduced mean value of 29.598% and 28.113%, respectively. At 3.794 GHz, the SM design successfully reduced the SAR values more than 60% for both 1 g and 10 g of tissue volume. The 10 g has the highest reduction percentage value of 70.33%, while the least value was recorded at 4.858 GHz with 8.45% in 1 g of tissue volume.

**Table 5:** SAR reduction values of 1 g of tissue volume with and without proposed metamaterial design.

Resonance frequency (GHz)	Without metamaterial design (W/kg)	With metamaterial design (W/kg)
1.246		
	0.557	0.411
3.052		
	0.404	0.313
3.794		
	1.816	0.705
4.858		
	0.832	0.762

**Table 6:** SAR reduction values of 10 g of tissue volume with and without proposed metamaterial design.

Resonance frequency (GHz)	Without metamaterial design (W/kg)	With metamaterial design (W/kg)
1.246		
	0.354	0.281
3.052		
	0.175	0.157
3.794		
	0.721	0.214
4.858		
	0.294	0.261

6 $$SAR=\frac{\sigma {(E}^{2})}{{m}_{p}}$$

## Parametric study

The simulation analysis of various parameters that carried out before producing optimised metamaterial design were discussed in this section. The performance comparison between these parametric studies is necessary to find closure to the proposed metamaterial design. These studies help to obtain specific parameters changing effects and how these findings do influence the research goal. Subsequently, few constraint variables were examined in this paper for instance, multiple square-shaped metamaterial design structures, array metamaterial design, several positions of the array cell inside the mobile phone, and various gaps between mobile phone and human head model. In the end, these numerical simulations assist to determine the superior design structure for electromagnetic absorption reduction applications.

### Unit cell analysis

**Figure 5:**
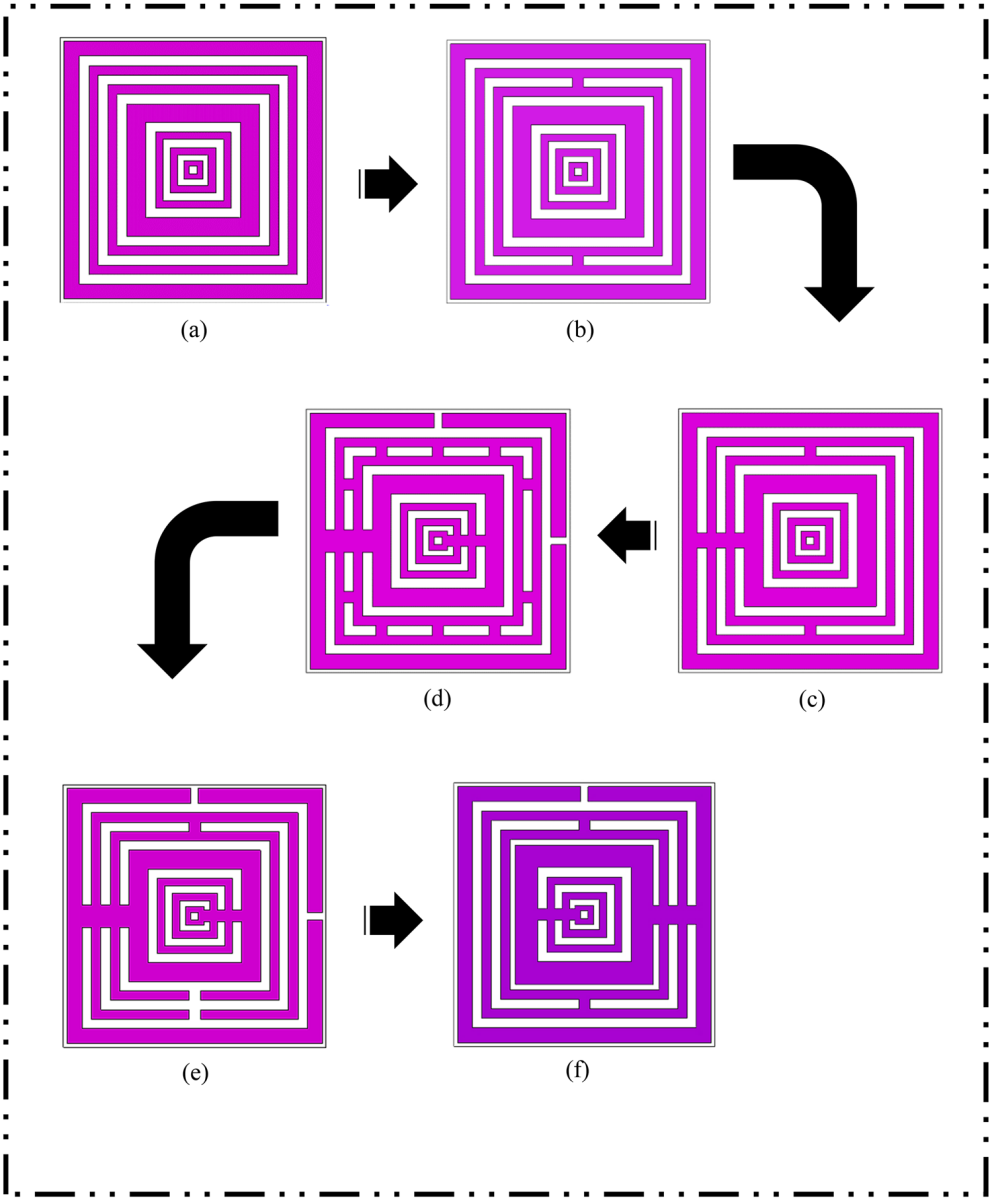
Various square-shaped metamaterial structures: (**a**) DS 1, (**b**) DS 2, (**c**) DS 3, (**d**) DS 4, (**e**) DS 5, (**f**) SM design.

During the preliminary analysis, we discovered that the square-shaped metamaterial design exhibits lower band resonance frequencies then the other structures, particularly circular design. This interesting outcome is worth discussing the fact that the square-shaped SRR generally induces an electromagnetic force around its structure. This structure allows the current to stream through one ring to another between the inner-ring spacing with the help of external magnetic fields. Furthermore, the square SRR metamaterial has higher current distribution than other structures and basically produces resonance frequencies at lower bands. It is notable that this type of composite structure has a supplementary capacitive coupling that helps to exhibit strong unique behaviours. For this analysis, a few types of square metamaterial design (DS) structures were adopted as demonstrated in Fig. 5a–f. The designs, DS 1 to DS 5 have similar dielectric substrate material and dimension, that is identical with the proposed metamaterial design. There were some slight differences in the design structures that presented in Fig. 5 but exhibits a major effect on the resonance frequencies. The connecting bar and subtraction bar were designed in particular ways for each metamaterial design in this analysis. Meanwhile, the trial and error method was utilised to identify the number of square SRR needed for this application field. It is important to highlight the fact that the number of square rings greatly influences the scattering parameters results. Alternatively, it could simply mean that the design construction underwent a few simulations with the identical method, but the designs were performed repeatedly until goods results were obtained.

**Figure 6:**
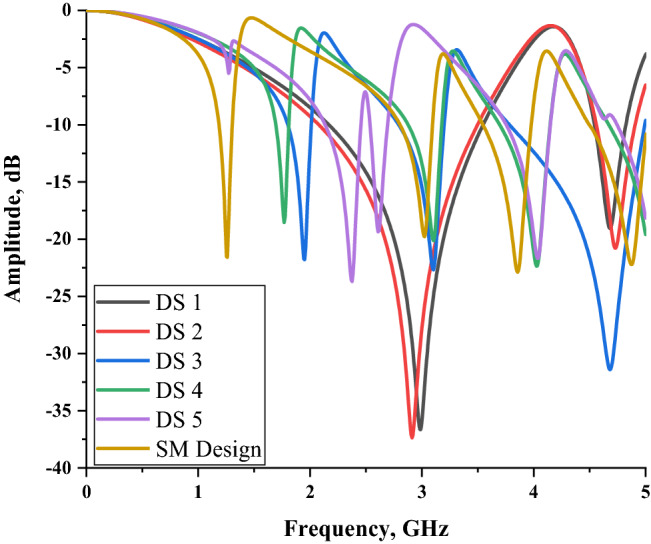
Transmission coefficient of all design structures.

The Fig. 6 demonstrates a heterogeneous transmission coefficient results for all metamaterial design structures. This section comprises six different unit cell designs comparison that manifest two double, three triple and one quadruple resonance frequencies. The preliminary study about the dimension of substrate material reveals that the smaller design structure shifts resonance bands toward higher frequencies. Meanwhile, the bigger dielectric substrate material did not meet the goal of this research paper. It has something to do with the fact that the latest telecommunication technology requires miniaturization aspects applied for any new designs or concepts. The main concern is the development of acceptable metamaterial design for the EM absorption reduction application. During the metamaterial construction process, we faced some issues in designing compact square-shaped metamaterial design. Therefore, the smallest possible substrate dimension material was utilised in this study to manifest lower frequency bands, which is 14 × 14 mm^2^. The first two designs have almost similar resonance frequencies with below − 15 dB magnitude values which fall in S- and C-bands, respectively. Following from these two designs, the next three designs in the Fig. 5 have the first resonance frequency shifted to a lower band. The connecting bar that was added in the left side of metamaterial structure as in the DS 3 design, has great impact on the first resonance frequency. Consequently, the initial resonance frequency of the design was shifted from S-band to L-band. Meanwhile, the small changes in the subsequent DS 3, DS 4 and SM designs cause an abrupt shifting occurred for the first resonance frequency. Furthermore, when the subtract bar was added at bottom structure of DS 5 instead of the connecting bar, the manifested first resonance frequency maintained at the S-band with slightly shifted to 2.375 GHz. Hence, from this standpoint, the SM design is considered as the optimised metamaterial structure for this research study.

### Array cell analysis

**Figure 7:**
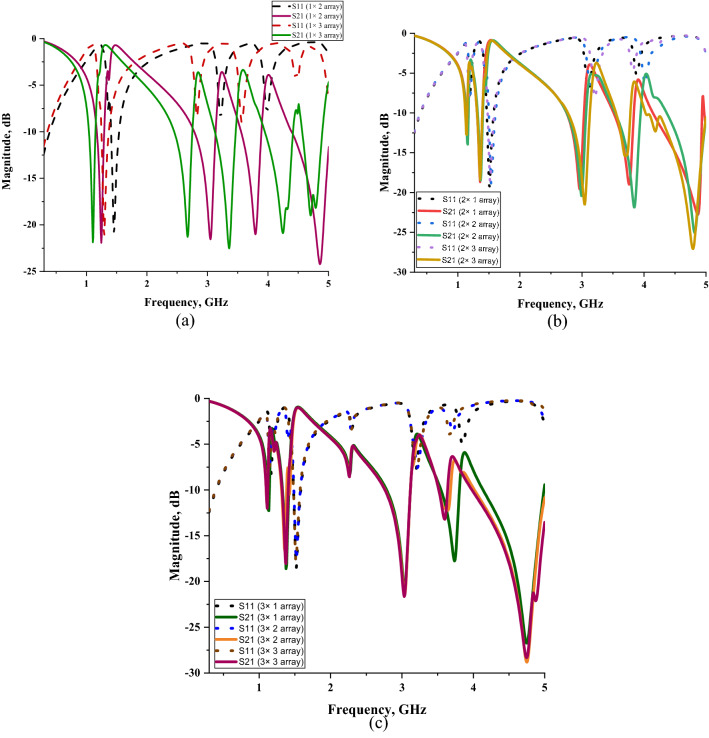
Comparison of reflection and transmission coefficient results of array metamaterials of: (**a**) one number row, (**b**) two number rows, (**c**) three number rows.

The unit cell generally does not work alone for industrial applications to produce extraordinary electromagnetic properties. Hence, data collections were conducted for a few sets of array cells (row × column), likely 1 × 2 (14 × 28 mm^2^), 1 × 3 (14 × 42 mm^2^), 2 × 1 (28 × 14 mm^2^), 2 × 2 (28 × 28 mm^2^), 2 × 3 (28 × 42 mm^2^), 3 × 2 (42 × 28 mm^2^), and 3 × 3 (42 × 42 mm^2^). The identical trial and error method was utilised in this analysis to discover the optimised array structure for the SAR reduction application. The selected array cells were numerically simulated by utilising CST software based on the simulation techniques described in “Numerical methods” section. These array cells analysis data was collected and illustrated in three different plots, namely one, two and three rows structure as shown in Fig. 7a–c. Each figure consists of at least two types of similar number of rows that demonstrate the transmission and reflection coefficient array metamaterial design, respectively. The one row array metamaterial design manifests quite similar transmission coefficient results when compared to the proposed unit cell design. Meanwhile, the results in Fig. 7b,c show clear evidence that the array cells exhibit five resonance frequencies but have moderate magnitude values. Furthermore, the array cells in these figures have less than one percentage of discrepancies occurred between them. Besides that, the increment of rows and columns did not seem to contribute a major effect on the transmission coefficient results. Nevertheless, during the selection of the array cell, the two and three rows were eliminated at the initial stage. Meanwhile the 1 × 2 array cell SM design has approximately less than 5% of inconsistency when compared with the unit cell design. This allows us to neglect the discrepancies between the two cells and take it as a viable option. The selection process of the array cell metamaterial has severe limitations and restrictions that need to be taken into consideration. The metamaterial structure controls the size constraint that becomes the key role in the latest technology applications. Hence, the compact size, 1 × 2 array cell metamaterial was selected because it is more appropriate and not irrelevant for the SAR reduction application.

### SAR reduction analysis

In general, the radio frequency energy will be transmitted to the surroundings when the mobile phone is in the operating mode. Thus, this energy can easily be absorbed by the head or any human body parts that are near to the device. Besides that, the latest 5G mobile network is new development to the world and precautions are necessary to avoid any unintended harmful consequences. Scientists around the world are investigating the effects of the radio frequency exposure from the 5G telecommunication network to humans. The SAR simulation was successfully carried out for all the resonance frequencies operating at the highest power level. Several distinct positions of metamaterial structure and phone model against voxel human head were analysed in this section. Typically, people have a unique way of holding the mobile phone and have different levels of radiation exposure. The outcomes of this study were collected and reviewed to discuss the handful of best positions in this paper.

#### Positions of array metamaterial design in mobile phone

**Figure 8:**
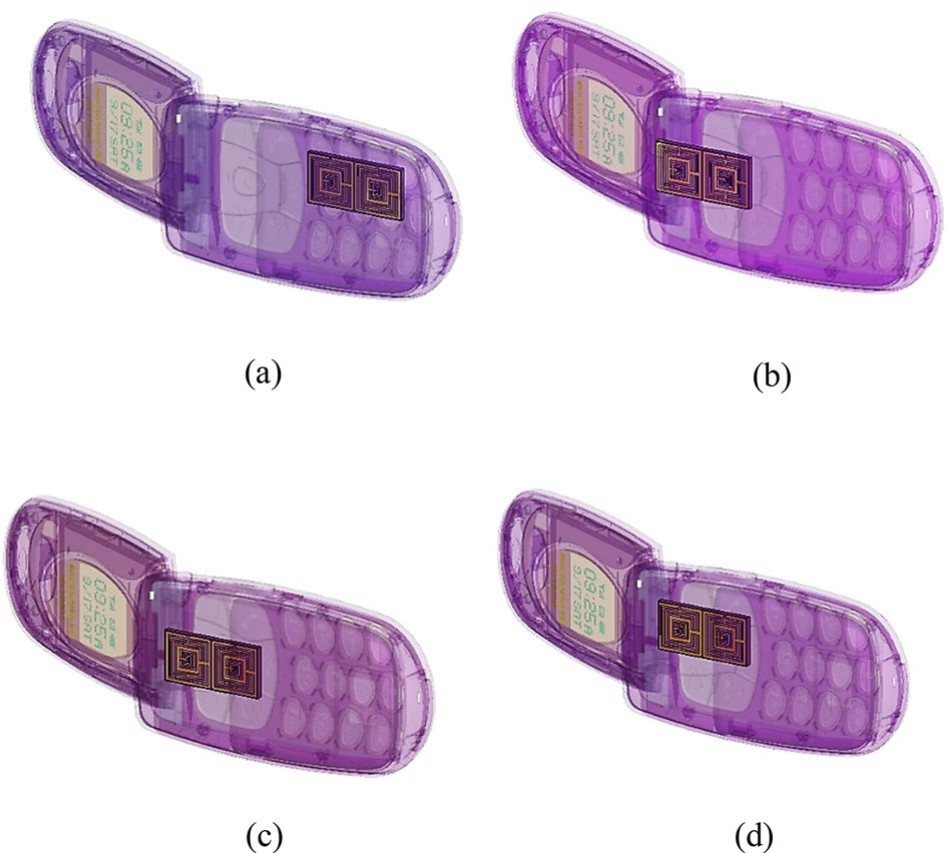
Placement of SM design in mobile phone: (**a**) position 1, (**b**) position 2, (**c**) position 3, (**d**) position 4.

Thereafter the selection of optimised array metamaterial structure, a few strategic positions of SM design inside the mobile phone were investigated. This analysis acts as a key role in identifying the enhanced SAR reduction values by placing the 1 × 2 array cell in various positions as illustrated in Fig. 8a–d. The 75% of array cells were positioned near to the mobile phone antenna and manifest discrete SAR reduction values. Initial study also reveals that the array cell is not necessarily placed alongside the antenna structure to exhibit enhanced performance. The trial and error method was utilised to recognise the suitable and best array cell placement. The performances of the diverse array positions were recorded and displayed as in Tables 7, 8, 9 and 10. The tables clearly indicate that, the SAR values of both 1 g and 10 g of tissue volumes for without and with metamaterial design proof the statement mentioned above, the relationship of distance between metamaterial to antenna. For over 1 g and 10 g of tissue volumes, Position 1 had the least SAR reduction percentage values for all the resonance frequencies. The first three array cell positions have at least two slightly higher SAR values for either 1 g or 10 g of tissue volumes when compared to results of the without array metamaterial structure. The sequential order of SAR percentage values that manifested by the array positions discussed in this section is arranged as Position 1, 3, 2, and 4. The highest recorded SAR percentage values fell in Position 4 with 61.16% and 70.33% for 1 g and 10 g of tissue volumes respectively. Although the array metamaterial structure is not necessarily placed very close to the mobile antenna, the Position 4 was placed below it with some distance. Although the distances between the array structure and antenna in Position 2 and 4 are slightly similar, the location of the structure is greatly influencing the outcome. Hence, the various SAR values were obtained through this analysis and caused by the placement of array metamaterial structure inside the mobile phone. In overall, the resonance frequency at 3.794 GHz has the highest reduction percentage in Position 4 when compared to rest of resonance frequencies. Meanwhile, at this frequency, other positions either have lower than or more zero percentage values. Hence, here we can conclude that the position of the metamaterial structure will influence the SAR reduction values. Therefore, through the trial and error method, the Position 4 exhibits better performance. The metamaterial structure does not necessarily place near to antenna or specific resonance frequency will manifest desired outcome. These two important factors proved in this analysis, where the Position 2 which is near to the mobile antenna, produce higher SAR value with metamaterial design. Furthermore, the highest recorded SAR reduction resonance frequency has lower than zero percentage values like in Position 1. These phenomena may occur because some reflection manifests at the interface and the entire radiofrequency energy is unable to be absorbed inside the proposed metamaterial. This causes an increment in the SAR values for several positions. Therefore, once finalised the resonance frequency from the array cell metamaterial simulation, the SAR reduction in this paper solely depends on the position of metamaterial inside the mobile phone. In conclusion, the Position 4 was selected because it manifested supreme and optimised SAR reduction values.

**Table 7:** Array metamaterial position 1.

Frequency (GHz)	1.246	3.052	3.794	4.858
Without 1 g	0.557	0.404	1.816	0.832
With 1 g	0.642	0.460	2.116	0.822
Percentage (%)	− 15.23	− 13.84	− 16.52	1.26
Without 10 g	0.354	0.175	0.721	0.294
With 10 g	0.431	0.171	0.887	0.280
Percentage (%)	− 21.47	2.51	− 23.04	4.78

**Table 8:** Array metamaterial position 2.

Frequency (GHz)	1.246	3.052	3.794	4.858
Without 1 g	0.557	0.404	1.816	0.832
With 1 g	0.383	0.469	1.544	0.751
Percentage (%)	31.25	− 15.94	15.00	9.84
Without 10 g	0.354	0.175	0.721	0.294
With 10 g	0.139	0.202	0.631	0.263
Percentage (%)	60.72	− 15.49	12.56	10.64

**Table 9:** Array metamaterial position 3.

Frequency (GHz)	1.246	3.052	3.794	4.858
Without 1 g	0.557	0.404	1.816	0.832
With 1 g	0.573	0.389	1.333	0.744
Percentage (%)	− 2.80	3.83	26.62	10.59
Without 10 g	0.354	0.175	0.721	0.294
With 10 g	0.386	0.192	0.542	0.255
Percentage (%)	− 9.03	− 10.02	24.79	13.23

**Table 10:** Array metamaterial position 4.

Frequency (GHz)	1.246	3.052	3.794	4.858
Without 1 g	0.557	0.404	1.816	0.832
With 1 g	0.411	0.313	0.705	0.762
Percentage (%)	26.22	22.56	61.16	8.45
Without 10 g	0.354	0.175	0.721	0.294
With 10 g	0.281	0.157	0.214	0.261
Percentage (%)	20.74	10.15	70.33	11.23

#### Mobile phone position from human head with various distances

**Figure 9:**
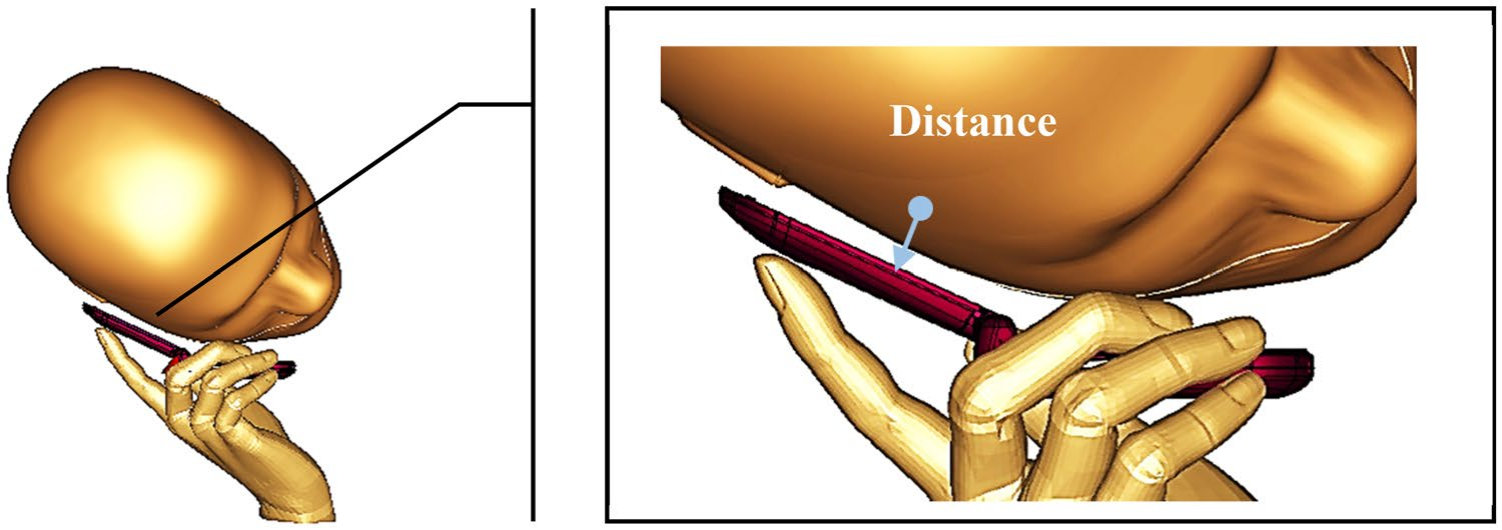
Mobile phone at 5 mm gap from the human head model.

The effect of various mobile phone distances from the human head model was investigated by calculating the SAR values for with SM design structure. The Fig. 9 illustrates the example of a mobile phone with 5 mm gap from the human head. The fundamental assumption is the relationship between mobile phone and head model also has significant effects on the SAR values. In spite of the fact that, longer the distance of a mobile phone from the human head then the SAR values that calculated will be reduced instantaneously. The SAR results of 5, 10, 15 and 20 mm distances for 1 g and 10 g of tissue volumes were collected and recorded with SAR contour head model in Tables 11 and 12. This comparison table reveals few critical details that help to understand the relationship. The highest SAR value recorded at 4.858 GHz in the primary distance, while the least occurred at 3.052 GHz in the 20 mm gap for 1 g and 10 g of tissue volumes, respectively. In the second resonance frequency of S-band, the metamaterial manifested least SAR value at initial distance of 0.097 W/kg for 10 g of tissue volume. In addition to that, at C-band the SM design exhibits a sharp drop of 0.14 W/kg that is from 5 to 10 mm gap for 1 g of tissue volumes. Nearly, all the SAR results have a gradual decline characteristic and proof the assumption stated above. A lower SAR value was achieved when the mobile phone moved far away from the human. A consistent finding was obtained between SM design and the 5 mm, 10 mm, 15 mm, and 20 mm distances among the phone model and the human head. Since people have their own way of holding the mobile phone, hence it is highly recommended to give spacing from the head during the usage.

**Table 11:** SAR values of 1 g of tissue volume for various mobile phone distance from human head.

Frequency (GHz)	5 mm	10 mm	15 mm	20 mm
1.246				
	0.287	0.202	0.153	0.114
3.052				
	0.187	0.098	0.087	0.071
3.794				
	0.294	0.194	0.175	0.144
4.858				
	0.436	0.296	0.220	0.197

**Table 12:** SAR values of 10 g of tissue volume for various mobile phone distance from human head.

Frequency (GHz)	5 mm	10 mm	15 mm	20 mm
1.246				
	0.204	0.146	0.112	0.086
3.052				
	0.097	0.051	0.045	0.038
3.794				
	0.121	0.088	0.072	0.061
4.858				
	0.162	0.118	0.091	0.081

## Comparison of previous SAR reduction studies

Table 13 represents the comparison of present SAR reduction research works with the proposed metamaterial design. The authors of all stated references claimed metamaterial structure only for EM absorption reduction application. Meanwhile, besides proposed metamaterial, the rest of previous reference papers that analysed here have similar double-negative electromagnetic characteristics. The electromagnetic properties of the man-made material can categorise into four types. A left-handed characteristic also commonly known as superior electromagnetic property of all time, was successfully declared in this paper with the proposed square-shaped metamaterial design. The left-handed metamaterial structure offers secondary advantages in other physical properties likely, heat conduction, strength, or elasticity. Besides that, left-handed metamaterial provided backward wave propagation support, that has anti-parallel wavenumber vectors to the transmitted power direction. Almost all the existing studies utilised copper material to design the metamaterial structure. This is due to the Table 11. SAR values of 1 g of tissue volume for various mobile phone distance from human head economical copper material that has high conductivity besides silver and gold. In other words, it can easily transmit signals without losing electricity along the way. Although the few previous studies show better SAR reduction values, they have few setbacks. These studies reveal that the left-handed metamaterial is usually problematic to obtain for all design structures. Nearly all the studies in Table 13, proposed a SRR metamaterial structure that manifests negative permeability and permittivity values. The arrangements of a specific number of rings and split between rings have great influence in the manifested outcome. As discussed earlier, the square-shaped metamaterial design manifests higher current distribution with lower resonance frequencies. In general, this design structure has an effective natural frequency which can be tuned by altering the arrangement of the rings. Hence, we specifically selected this shape to apply in the mobile application fields. Concern about the miniaturization of electronic products and devices have become a new trend in the scientific community. The references^34–37^ have bigger array cells that become a major limitation for the SAR reduction application. Because of this potential restriction, we treat the size miniaturization of the array cell by giving considerable importance during the construction of metamaterial design. Furthermore, to our knowledge, limited SAR reduction studies have been examined for the 5G Mobile Network radiation effects. Besides that, the metamaterial designs that proposed in the literature reviews have either single or double resonance frequencies only. Hence, a novel compact square-shaped design structure was proposed in this research study, which is assigned to the fifth-generation technology standard for cellular networks. The proposed metamaterial structure is designed in a distinct way with a specific number of square rings and connected by a rectangular copper bar at few ring structures. Moreover, this design also exhibits quadruple resonance frequencies and at the same time has acceptable SAR reduction percentage values. Therefore, this study of multi-resonance frequencies in sub-6 frequency range for electromagnetic absorption reduction with defined compact square-shaped metamaterial structure become the novelty of this paper.

**Table 13:** A comparison between proposed metamaterial design and previous studies.

Referencess	Dimension (mm)	Characteristic	Resonance frequencies	Band	Reduction (%)
^34^	AC: 44.4 × 33.3	Double-negative	900 and 1800 MHz	Double	1 g: 43 and 44
^35^	AC: 187 × 117	Double-negative	2100 MHz	Single	1 g: 35.93
^36^	AC: 54.72 × 39	Double-negative	900 and 1800 MHz	Double	1 g: 40 and 49
^37^	AC: 36 × 36	Double-negative	900 and 1800 MHz	Double	1 g: 14.23 and 15.3
Proposed	AC: 14 × 28	Left-handed	1.246, 3.052, 3.794, and 4.858 GHz	Quadruple	1 g: 26.22 22.56, 61.16 and 8.45

## Conclusion

The authors concluded that the attachment of square-shaped metamaterial inside the mobile phone model helps to reduce the SAR values that are computed by utilising CST software. Since the new telecommunication network upon us and the radiation exposure becomes the main concern among the scientific community. Furthermore, the consideration about radiation exposure to humans has increased tremendously in the past few decades due to rapid growth in the usage of wireless communication devices among people. Therefore, current research fields were investigating the approaches to lower the radiation effect that was exposed from the mobile phone. The phantom model known as Specific Anthropomorphic Mannequin was exported from CST and was utilised to conduct numerical simulation of SAR values. Significant four resonance frequencies with acceptable magnitude values were exhibited by the proposed metamaterial precisely at 1.246, 3.052, 3.794, and 4.858 GHz. Overall, three resonance bands were analysed through this simulation. Each band at least has a single resonance frequency while, S-band has dual resonance behaviour. The validation process for the electromagnetic properties of simulation results was performed by utilising VNA measurement kid. Broadly translated these findings indicate that the discrepancy between the simulated and measured results was highly neglectable because it has less than 0.15 GHz differences. Moreover, the least SAR value recorded was more than 8% at last resonance frequency for 1 g of tissue volume. The SAR values have inconsistent rise and fall throughout all the resonance frequencies for both 1 g and 10 g of tissue volumes. The manifested triple resonance bands can be applied in a broad range of applications such as satellite applications, whether radar, wireless headphones (Bluetooth), surface ship radar, garage door openers, keyless vehicle locks, wireless networking (Wi-Fi), etc. In a nutshell, the proposed SM design structure successfully achieved the goal of this research work by reducing the SAR values and possesses unique electromagnetic properties.
